# Mono-(2-Ethylhexyl) Phthalate Promotes Pro-Labor Gene Expression in the Human Placenta

**DOI:** 10.1371/journal.pone.0147013

**Published:** 2016-01-11

**Authors:** Ximi K. Wang, Monica Agarwal, Nataliya Parobchak, Alex Rosen, Anna M. Vetrano, Aarthi Srinivasan, Bingbing Wang, Todd Rosen

**Affiliations:** 1 Biomedical Sciences Graduate Program, Rutgers Robert Wood Johnson Medical School, Piscataway, New Jersey, United States of America; 2 Department of Obstetrics, Gynecology, and Reproductive Sciences, Rutgers Robert Wood Johnson Medical School, New Brunswick, New Jersey, United States of America; 3 Department of Pediatrics, Rutgers Robert Wood Johnson Medical School, New Brunswick, New Jersey, United States of America; Qingdao Agricultural University, CHINA

## Abstract

Women exposed to phthalates during pregnancy are at increased risk for delivering preterm, but the mechanism behind this relationship is unknown. Placental corticotropin-releasing hormone (CRH) and cyclooxygenase-2 (COX-2) are key mediators of parturition and are regulated by the non-canonical NF-kB (RelB/p52) signaling pathway. In this study, we demonstrate that one of the major phthalate metabolites, mono-(2-ethylhexyl)-phthalate (MEHP), increased CRH and COX-2 mRNA and protein abundance in a dose-dependent manner in primary cultures of cytotrophoblast. This was coupled with an increase in nuclear import of RelB/p52 and its association with the *CRH* and *COX-2* promoters. Silencing of NF-kB inducing kinase, a central signaling component of the non-canonical NF-kB pathway, blocked MEHP-induced upregulation of CRH and COX-2. These results suggest a potential mechanism mediated by RelB/p52 by which phthalates could prematurely induce pro-labor gene activity and lead to preterm birth.

## Introduction

Preterm birth is the leading cause of neonatal mortality worldwide and affects close to 12% of all pregnancies in the United States[1, 2]. Prematurity can lead to adverse health effects lasting well into childhood and adulthood [3–5]. The potential effects of exposure to environmental toxins on spontaneous preterm birth are an area of active investigation.

Phthalates are a pervasive class of chemical plasticizers found in numerous consumer products worldwide. Human health effects from phthalates at low environmental exposures are unknown, but recent studies have linked phthalate exposure to preterm labor [6] [7, 8]. Diethylhexyl phthalate (DEHP), commonly used in the manufacturing of polyvinyl chloride (PVC), is an additive that is particularly concerning. High concentrations of mono-2-ethylhexyl phthalate (MEHP), the active metabolite of DEHP, in umbilical cord blood was associated with decreased gestational length[9, 10]. Furthermore, a recent nested case-control study found that women exposed to phthalates, including DEHP and MEHP, have significantly increased odds of delivering preterm [11].

Despite a number of correlation studies, there is only limited information about the possible mechanisms underlying a link between maternal phthalate exposure and preterm labor. A study by Vetrano et al. demonstrated that MEHP exerts pro-inflammatory effects in neonatal neutrophils, suggesting one pathway that could be involved in phthalate-initiated preterm birth [12]. Tetz et al. found that phthalates stimulated COX-2 mediated prostaglandin E-2 synthesis in human placental macrophages and this could constitute another mechanism of toxicant-associated preterm birth [13].

Placental CRH, which is positively regulated by glucocorticoid [14], may be part of a clock that governs the length of gestation in humans [15]. Prostaglandins (PGs) generated by cyclooxygenase-2 (COX-2) also play an important role in initiation of human labor [16]. Recent studies in our laboratory have demonstrated that CRH and COX-2 of human placental origin are positively regulated by RelB/p52 induced by the non-canonical nuclear factor- kappa B (NF-kB) pathway [17, 18]. Our previous work suggests that the non-canonical NF-kB pathway may play a central role in governing the clock that determines the length of pregnancy. We set out to determine whether phthalates can also stimulate the non-canonical NF-kB pathway, and in turn, activate pro-labor genes in a model of placental function.

In the present study, we found that MEHP stimulated expression of CRH and COX-2 in a dose-dependent manner in primary cultures of human syncytiotrophoblast. We also demonstrated that MEHP exposure increased nuclear translocation of RelB/p52 heterodimers and its association with the *CRH* and *COX-2* promoters. Knockdown of NF-kB inducing kinase (NIK), a key activator of the non-canonical NF-kB pathway, inhibited MEHP-induced up-regulation of *CRH* and *COX-2*. These results suggest a molecular mechanism linking phthalates to premature activation of the pro-labor genes *CRH* and *COX-2*, and potentially preterm labor in humans.

## Materials and Methods

### Study approval

We collected term human placentas (from 37 to 41 weeks gestational age) from healthy women following Caesarean section without labor, after patients signed a written informed consent form. This study protocol was approved by the Institutional Review Board of Rutgers University.

### Purification of primary cytotrophoblasts

Placental cytotrophoblasts were purified as previously described[17, 19], cultured in DMEM/F-12 (Sigma-Aldrich, MO) with 10% fetal bovine serum (FBS) (Life Technologies, NY), and maintained at 37°C and 5% CO2 for 24 hours before further analysis.

### Cell viability assay

Primary cytotrophoblast were treated with MEHP at concentrations as indicated for 24 hrs. Cell viability was determined with use of trypan blue exclusion assay (ThermoFisher Scientific, NY) according to the manufacturer’s specifications. The percentage of viable cells was calculated by the formula: [1.00 –(number of blue cells/number of total cells)] × 100.

### Western blot

Whole cell lysates were resolved on sodium dodecyl sulfate polyacrylamide gel electrophores (SDS-10% PAGE) and transferred onto polyvinyl difluoride (PVDF) membranes (Bio-Rad Laboratories, Hercules, CA). Membranes were blocked in 5% nonfat milk powder-phosphate buffered saline (PBS) for 1 hour, and then incubated with individual antibodies at 4°C overnight. Membranes were then incubated with the appropriate horseradish peroxidase-conjugated secondary antibodies at a 1:5,000 dilution in 1% nonfat milk powder- PBS-Tween 20 PBST, and developed by Immuno-Star horseradish peroxidase HRP substrate (Bio-Rad Laboratories). Semi-quantitative analysis was accomplished with use of Multi-Analyst software (Bio-Rad, CA).

### Reverse transcription quantitative PCR (RT-qPCR)

Total RNAs were prepared with use of the Trizol method (Invitrogen). Complementary DNA (cDNA) synthesis was completed by a mixture of oligo-dT and the random primer method with use of the GoScript reverse transcription system (Promega, Madison, WI). The following primers were used for quantitative analysis with SYBR green dye (QIAGEN) in real-time PCR: CRH, forward, 5′- GCAGTTAGCACAGCAAGCTCAC-3′, reverse, 5′-CAAATGGCATAAGAGCAGCG-3′; COX-2, forward, 5′-TGAGCATCTACGGTTTGCTG-3′, reverse, 5′-TGCTTGTCTGGAACAACTGC-3′; NIK, forward, 5’-CAAGCCTCTGAAGGAACCAG-3’, reverse, 5’-AGGGATGAGGCAGTCTGCTA-3’, and GAPDH, forward, 5′-CTCCCGCTTCGCTCTCTG-3′, reverse, 5′- CTGGCGACGCAAAAGAAG -3′.

### Immunofluorescence (IF) staining

Primary cytotrophoblast cells were fixed following the 48-hr exposure period with 4% paraformaldehyde in PBS and permeabilized for 10 minutes in 0.5% Triton X-100 in PBS at room temperature. Cells were then washed with PBS and incubated with appropriate antibodies overnight at 4°C. Cells were washed with PBS containing 0.1% bovine serum albumin (BSA) and incubated with fluorophore-conjugated secondary antibodies (Alexa Fluor 488 goat anti-rabbit IgG and Alexa Fluor 532 goat anti-mouse, Invitrogen) for 1 hour at room temperature. The cells were then counterstained with 4',6-diamidino-2-phenylindole DAPI (Invitrogen) prior to being visualized under a fluorescence microscope (Nikon, Japan). We quantified IF staining by calculating the ratio of the cells with nuclear co-localization of RelB/p52 to the total number of cells per field, and obtained the average from three random fields.

### Chromatin Immunoprecipitation (ChIP)

ChIP was performed as detailed in our previous studies[17, 19]. Fold enrichment was calculated with the method of cycle threshold in the formula of 2-^ΔΔCT^. The primers (forward/reverse) used for the amplicon to CRH or COX-2 promoter encompassing the NF-kB enhancer were 5′-GGCCTTTCATAGTAAGAGGTCAA-3′/5′- TCTCACATCCAATTATATCAACAGA-3′ or 5′-AACATAAAACATGTCAGCCTTTCTTAACC-3′/5′-GTTTCTTTCCGCCTTTTCGTACC-3′, respectively. Control primers (forward/reverse) for a non-binding site (a human DNA alpha satellite) were 5′-TCTCAGAATCTTCCTTTTGATGTG-3′/5′-CCAGTTGCAGATCCTACAAAGA-3′.

### Gene Silencing

Freshly prepared human cytotrophoblast cells (3 × 10^6^ cells/well) were plated in 6-well culture plates in 3 mL of Dulbecco’s modified Eagle's medium (DMEM)/F-12 containing 10% FBS. After being maintained in culture for 24 hrs, each well was transfected with the complex of FlexTube small interfering RNA (siRNA) (Qiagen, Valencia, CA) targeting NIK and 3 uL of Lipofectamine 2000 (Invitrogen), prepared following manufacturer’s instructions with a final concentration of siRNA as indicated. A non-targeting siRNA or scramble RNA was used as a negative control with target sequences: 5′-AACAGUCGCGUUGUCGACUGGUU-3′. At 48-hr after exposure to the targeting or non-targeting siRNA, whole-cell lysates were prepared for Western blotting.

### Statistical Analysis

Each experiment was repeated in a minimum of three times. Data (bars) are presented as the means ± S.D. We used the Student *t* test (one-tailed) to compare the control with each individual test group. p < 0.05 was considered statistically significant.

## Results

### MEHP exposure leads to upregulation of CRH and COX-2 in the human placenta

**Fig 1:**
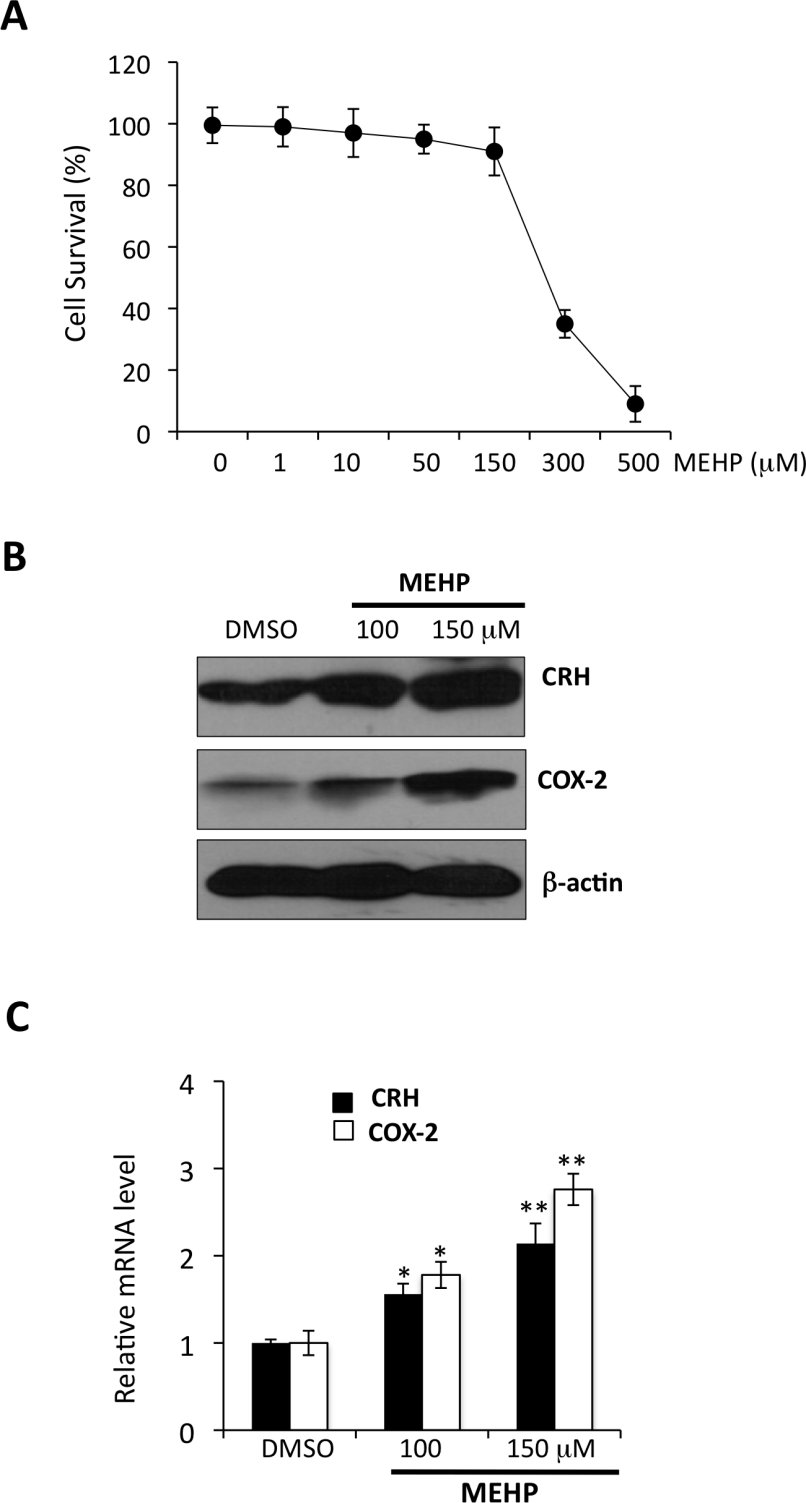
MEHP upregulates expression of CRH and COX-2 in primary cultures of human cytotrophoblast. Primary cultures of term cytotrophoblast were exposed to different concentrations of MEHP for 24 hours with DMSO as the vehicle control. (**A**) Trypan blue exclusion test of cell viability for primary cytototrophoblast exposed to MEHP at concentrations as indicated (N = 3 independent experiments). (**B**) Representative gel patterns and analysis in whole cell lysates from three independent term placentas by Western blot assay with use of antibody as indicated. (**C**) Total RNAs were extracted and RT-qPCR was performed for assessment of CRH and COX-2 mRNA levels with normalization to GAPDH mRNA level. Bars represent the average of relative mRNA levels and error bars represent standard deviation from experiments performed in three independent term placentas. * p < 0.05; ** p < 0.01.

We characterized cytotoxic effects of MEHP on viability of primary cytotrophoblast. Primary cells were incubated with MEHP at a serial dilution of 10 to 500 uM for 24 hrs. We observed that the average of survival rates of cytotrophoblast exposed to MEHP at 150, 300, and 500 uM from three independent experiments was > 90%, <40%, and <10%, respectively (Fig 1A). We tested effects of MEHP on expression of CRH and COX-2 at concentrations of 1, 10 50, 100, 150 uM, and found no significant effects with concentrations from 1 to 50 uM (data not shown). We conducted the remainder of the experiments with MEHP at concentrations of 100 and 150 uM. These concentrations are also consistent with those observed by Tetz et al. showing that MEHP at from 90 to 180 uM had significant effects on induction of COX-2 in THP-1 cells, a human macrophage-like cell line [13]. As shown in Fig 1B and 1C, MEHP increased abundance of CRH and COX-2 mRNA and proteins levels in a dose-dependent manner.

### MEHP exposure induces nuclear translocation of RelB/NF-kB2 (p52)

**Fig 2:**
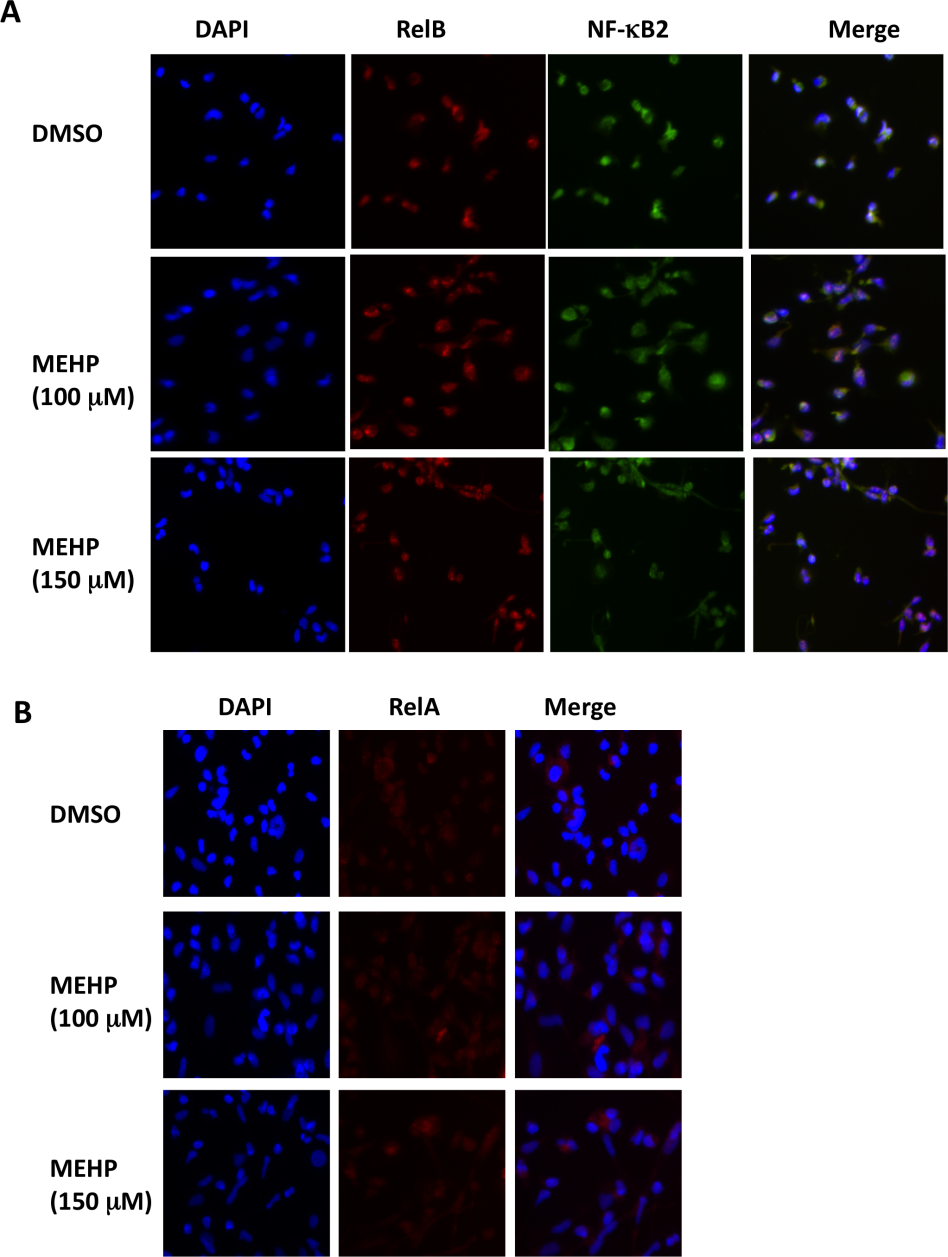
MEPH exposure increases nuclear RelB/p52. Primary term cytotrophoblast were exposed to MEHP for 24 hours at concentrations as indicated with DMSO as the vehicle control. Immunofluorescence (IF) staining was performed for direct visualization of intracellular distribution of RelB/p52 (**A**) and RelA (**B**), as detailed in the Materials and Methods. These images represent data obtained from three independent placentas. Original magnification, 200Χ.

Recently, we have shown that the non-canonical NF-kB pathway induces RelB/NF-kB2 heterodimer nuclear localization which, in turn, up-regulates *CRH* and *COX-2* in the human placenta[17, 19]. NF-kB proteins including RelB/NF-kB2 are normally sequestered in the cytoplasm. NF-kB2 is translated as the precursor peptide p100 that acts as an NF-kB inhibitor. Proteasome-mediated processing of p100 produces the active protein p52 which results in translocation of RelB/p52 into the nucleus of the cell where it affects gene transcription[20]. We used immunoflourescence staining to investigate the effects of MEHP on nuclear localization of RelB/p52. In Fig 2A, we demonstrate that MEHP exposure significantly increased nuclear presence of both RelB and p52. Quantification analysis showed that the ratio (average ± S.D.) of nuclearly co-localized RelB/p52 was 52.6 ± 5.7% and 58. 4 ± 6.2% for 100 and 150 uM of MEHP, respectively, compared to 32.6 ± 4.5% for the DMSO control (p < 0.01). In contrast, MEHP treatment exerted no noticeable effect on nuclear RelA (Fig 2B), an end product of the canonical NF-kB signaling pathway. These results indicate that MEHP-mediated up-regulation of *CRH* and *COX-2* could potentially be induced by nuclear localization of RelB/p52.

### MEHP exposure promotes association of RelB/p52 with *CRH* and *COX-2*

**Fig 3:**
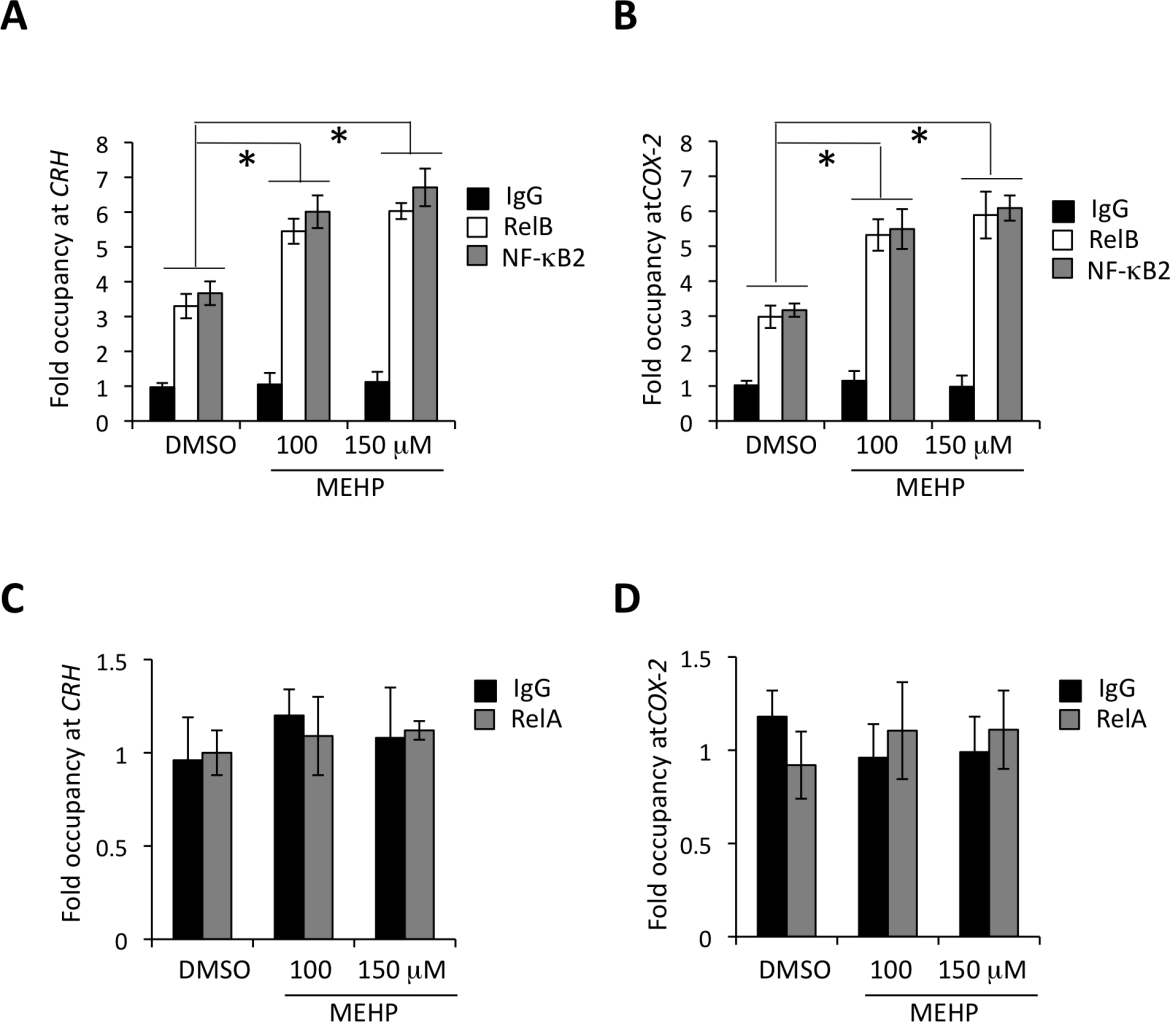
The effects of MEHP exposure on interaction of RelB/p52 with the *CRH/COX-2* gene promoters. Term cytotrophoblast were exposed to MEHP for 24 hrs at concentrations as indicated. ChIP assays were performed to determine occupancy of RelB/p52 at *CRH* (**A**) and *COX-2* (**B**), and also occupancy of RelA at the *CRH* (**C**) and *COX-2* (**D**) gene promoter. Fold enrichment was derived with normalization to a human DNA a satellite, a non-coding DNA sequence to which no transcriptional factors should bind. Rabbit IgG was used as a non-specific control. The bars indicate the average of fold enrichment with error bars representing the standard deviation from three independent experiments. * p < 0.01.

NF-kB proteins contain a highly homologous Rel domain that is responsible for protein-DNA (NF-kB enhancer) interaction and hetero- or homo-dimerization[21]. We have previously shown a specific interaction between the kB response elements at the *CRH* and *COX-2* promoters and RelB/p52 in the human placenta[17, 19]. Consistent with our previous data, *CRH* or *COX-2* promoter was almost equally occupied by RelB and p52 in cytotrophoblasts, approximately 3-fold over the non-specific control (Fig 3A and 3B). Primary cytotrophoblast, when exposed to MEHP, demonstrated > 2-fold increase of RelB/p52 occupancy at the *CRH* and *COX-2* promoters. There was no interaction found between RelA and *CRH* or *COX-2* under the same conditions (Fig 3C and 3D).

### Depletion of NIK attenuates MEHP-induced expression of pro-labor genes

**Fig 4:**
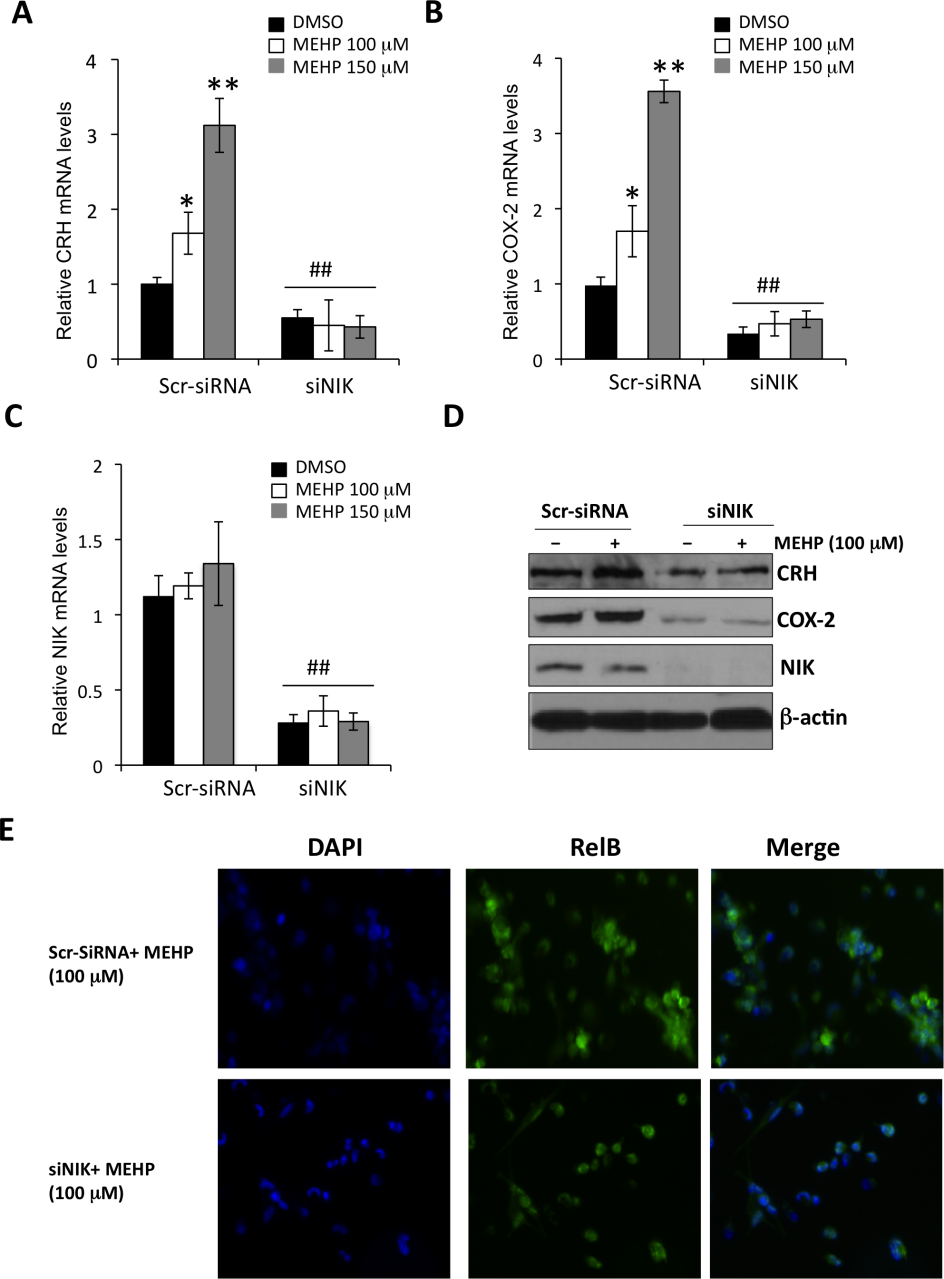
NIK knockdown attenuates MEHP-induced upregulation of *CRH* and *COX-2* in the human placenta. Primary term cytotrophoblast were incubated with siRNAs targeting NIK (siNIK) for 24 hrs, and then treated with MEHP for an additional 24 hrs. Scramble siRNA (Scr-siRNA) was used as the non-targeting control. Total RNAs were extracted and RT-qPCR was performed to determine mRNA levels of CRH (**A**), COX-2 (**B**), or NIK (**C**) (N = 3 independent experiments). * p < 0.05 (compared to DMSO). ** p< 0.01 (compared to DMSO). ## p < 0.01 (compared to Scr-siRNA). (**D**) Representative gel pattern of Western blot analysis on whole cell lysates from three independent experiments. (**E**) IF staining was performed to directly assess intracellular distribution of RelB. These images represent data obtained from three independent placentas. Original magnification, 200Χ.

NIK is a central signaling component of the non-canonical NF-kB signaling pathway. NIK-induced p100 processing acts to both generate p52 and stimulate import of RelB/p52 heterodimers into the cell nucleus[20, 22]. We sought to dissect the stages of the non-canonical NF-kB pathway that MEHP targets in the human placenta. We found that knockdown of NIK inhibited MEHP-induced upregulation of *CRH* and *COX-2* (Fig 4A to 4D). No significant change was observed for NIK mRNA levels by RT-qPCR (Fig 4C), or protein levels by semi-quantitative analysis upon MEHP treatment (1.12 ± 0.15 in lane 1 v.s. 0.95 ± 0.21 in lane 2 at relative protein levels of NIK, p > 0.05) (Fig 4D). However, nuclear RelB was significantly reduced by depletion of NIK (53.4 ± 7.8% with control siRNA v.s 12. 5 ± 6.2% with NIK siRNA, p < 0.01) (Fig 4E). Together, these results suggest that MEHP agonistic effects on pro-labor genes are dependent on activity of NIK.

## Discussion

We have previously demonstrated that RelB/p52, activated by the non-canonical NF-kB pathway, regulates the pro-labor genes CRH and *COX-2* in the human placenta[17–19, 23]. Both CRH and prostaglandins produced by COX-2 play important roles in initiating human parturition [24]. In a previous study, MEHP was shown to increase levels of COX-2 in human placental cells[25]. In the present study, we also demonstrated that phthalates significantly increased abundance of *COX-2* and *CRH* in cytotrophoblast cultured from term human placenta. We further found that MEHP increased nuclear localization of RelB and p52, the effector proteins of the non-canonical NF-kB signaling pathway. Down-regulation of CRH and COX-2 via knockdown of NIK in cytotrophoblast provided additional evidence that the non-canonical NF-kB pathway is activated by phthalates and could impact the timing of labor.

In 2003, researchers from the National Health and Nutrition Examination Survey (NHANES) published that more than 75% of urine samples from subjects across the United States contained detectable phthalate levels. Levels of certain phthalates were higher in women than men. Non-Hispanic blacks, the group at highest risk for preterm birth had significantly higher concentrations of phthalates than did Mexican-Americans and non-Hispanic whites [26]. The high prevalence of phthalate exposure in pregnant women, observational studies linking phthalates to preterm birth, and plausible biologic mechanisms that could underlie this association make this a topic worthy of further investigation.

Average human umbilical cord concentrations of MEHP have ranged widely in published studies. Latini and colleagues found that average MEHP levels were 1.8 s 2.2 uM, with a range of 0–10.6 uM in an Italian population[9]. Working in China, Li and colleagues found that average MEHP umbilical cord concentrations were approximately 5.7 uM [27], but Lin and colleagues found higher levels that averaged 35 uM[28]. The concentrations of MEHP we employed *in vitro* were higher than those found in exposed human populations, but the doses of 100 and 150 uM were not toxic to cytotrophoblast, which suggests that our results are not artifactual. Our results are consistent with the recent study by Tetz and colleagues showing that significant effects were observed with MEHP concentrations ranging from 90 to 180 uM, but not for 10 to 90 uM, for induction of COX-2 in human placental macrophages and human macrophage-like cell line THP-1 cells[13]. It is possible that prolonged exposure to lower phthalate levels over the course of a long pregnancy (as opposed to 24 hours of cell culture) could have a clinically significant effect on the placental clock. This idea will need to be tested in an appropriate animal model in future studies. Nonetheless, the mechanism we have described here provides a plausible link between the observed association of maternal phthalate exposure and preterm birth.

Future research could further elucidate precisely how phthalates induce activation of non-canonical NF-kB signaling in the human placenta. NIK is known to be a signal integrator and central component of the non-canonical NF-kB pathway. It is generally true that intracellular accumulation of NIK is necessary and sufficient for p100 phosphorylation[20, 22]. Under normal circumstances, NIK is inactivated by the complex consisting of multi-subunit NIK ubiquitin ligases, which mediates constant ubiquitination followed by proteasomal degradation of NIK[29]. Activation of NIK is mediated by ligand cross-linking to a subset of the TNF receptor superfamily, including BAFFR, CD40, LTbR, and RANK, which triggers degradation of this ubiquitin ligase to stabilize NIK levels. NIK knockdown was found to prevent MEHP-induced nuclear translocation of RelB and expression of CRH and COX-2 in this study, consistent with that NIK is an upstream regulator of RelB/p52. However, we observed that MEHP treatment had no significant effects on the abundance of NIK. As a result, MEHP exposure-induced activation of RelB/p52, and in turn, CRH and COX-2, is not mediated by increased accumulation of NIK but likely by its post-translational modification(s). Indeed, it has been previously shown that ubiquitination of NIK at K63 by zinc finger protein 91 (ZFP91) promotes catalytic activity of NIK in processing of p100 to p52[30]. In addition, phosphorylation of NIK by its downstream kinase, IKKa, in a feedback mechanism can prevent accumulation of NIK to keep it at a steady level[31]. Further molecular characterization of relation of MEHP treatment and post-translational regulation of NIK could be our future directions.

These data add a novel mechanism to previously proposed ones for phthalate-preterm birth. It has been shown that phthalates have adverse effects in pregnant women in association with the marked increase in oxidative stress biomarkers[32, 33]. Concentrations of the oxidative stress biomarker 8-isoprostane were 50% higher in the urine of pregnant women exposed to phthalates when compared to controls. High 8-isoprostane levels in both plasma and urine are associated with spontaneous preterm birth and other complications such as preeclampsia and low-birth-weight neonates[34]. Because phthalates are also associated with inflammation, another potential cause of preterm birth, it is possible that there are multiple pathways by which phthalates negatively impact pregnancy outcomes.
